# TCF7/SNAI2/miR-4306 feedback loop promotes hypertrophy of ligamentum flavum

**DOI:** 10.1186/s12967-022-03677-0

**Published:** 2022-10-12

**Authors:** Yang Duan, Jianjun Li, Sujun Qiu, Songjia Ni, Yanlin Cao

**Affiliations:** 1 Department of Spine Surgery, Zhujiang Hospital, Southern Medical University, Guangzhou, China; 2 Department of Orthopaedic Trauma, Zhujiang Hospital, Southern Medical University, Guangzhou, China

**Keywords:** Ligamentum flavum hypertrophy, TCF7, SNAI2, miR-4306

## Abstract

**Background:**

Hypertrophy of ligamentum flavum (HLF) is the mainly cause of lumbar spinal stenosis (LSS), but the precise mechanism of HLF formation has not been fully elucidated. Emerging evidence indicates that transcription factor 7 (TCF7) is the key downstream functional molecule of Wnt/β-catenin signaling, which participated in regulating multiple biological processes. However, the role and underlying mechanism of TCF7 in HLF is still unclear.

**Methods:**

We used mRNAs sequencing analysis of human LF and subsequent confirmation with RT-qPCR, western blot and immunohistochemistry to identified the TCF7 in HLF tissues and cells. Then effect of TCF7 on HLF progression was investigated both in vitro and in vivo. Mechanically, chromatin immunoprecipitation, dual-luciferase reporter assays, and rescue experiments were used to validate the regulation of TCF7/SNAI2/miR-4306 feedback loop.

**Results:**

Our results identified for first time that the TCF7 expression was obviously elevated in HLF tissues and cells compared with control, and also found that TCF7 expression had significant positive correlation with LF thickness and fibrosis score. Notably, TCF7 inhibition suppressed the hyper-proliferation and fibrosis phenotype of HLF cells in vitro and ameliorated progression of HLF in mice in vivo, whereas TCF7 overexpression promoted hyper-proliferation and fibrosis phenotype of HLF cells in vitro. Our data further revealed that TCF7 interacted with SNAI2 promoter to transactivated the SNAI2 expression, thereby promoting hyper-proliferation and fibrosis phenotype of HLF cells in vitro. Furthermore, miR-4036 negatively regulated by SNAI2 could negatively feedback regulate TCF7 expression by directly binding to TCF7 mRNA 3’-UTR, thus inhibiting the hyper-proliferation and fibrosis phenotype of HLF cells in vitro.

**Conclusions:**

Our study demonstrated that TCF7 inhibition could suppress HLF formation by modulating TCF7/SNAI2/miR-4306 feedback loop, which might be considered as a novel potential therapeutic target for HLF.

**Supplementary Information:**

The online version contains supplementary material available at 10.1186/s12967-022-03677-0.

## Introduction

Lumbar spinal canal stenosis (LSCS) is the most common spinal disease in elderly patients [41], and hypertrophy of ligamentum flavum (HLF) is considered to be a major cause of LSCS [27, 42]. There is currently no particularly effective conservative treatment to delay or reverse the HLF-induced LSCS except surgical decompression. Surgical removal of the hypertrophic ligamentum flavum can achieve spinal decompression, but not only the risks and postoperative complications of surgery are particularly prominent but also many patients with underlying diseases (such as diabetes, hypertension and hyperlipidemia) are intolerant to surgery [21, 37, 43]. Although fibrosis have been proved to be the key pathological feature of HLF, the precise mechanism of the pathology of LF fibrosis has not been fully elucidated [32]. Therefore, investigating molecular pathways associated with LF fibrosis will provide insight into HLF mechanisms and identify novel targets for prevention and treatment of HLF-induced LSCS.

Transcription factor is a DNA binding proteins that can recognize specific DNA sequences to activate or inhibit gene transcription [15]. Studies have proved that abnormal expression of transcription factor is closely related to the occurrence and development of many human diseases [7, 29]. The TCF/LEF transcription factor family can bind to the WRE region within the promoter sequence or enhancer sequence of the target gene, thereby promoting or inhibiting transcription of the target gene [14]. Transcription factor 7 (TCF7, also known as TCF1) is one of the members of the TCF/LEF transcription factor family and participates in the transcriptional regulation of Wnt/β-catenin signal [1, 3]. Emerging evidence has shown that TCF7 is involved in tumorigenesis and development [2, 35, 44], sepsis-induced renal injury [39], heart development [40], and cardiac hypertrophy [26]. However, the roles and underlying mechanism of TCF7 in HLF have not been clarified.

Extensive evidence has shown that TCF7 is negatively regulated by microRNAs (miRNAs) [12, 28]. MiRNAs is a class of non-coding single-stranded RNA molecules with a length of about 19–25 nucleotides encoded by endogenous genes, which participate in the regulation of post-transcriptional gene expression in plants and animals [19]. Current studies have been demonstrated that dysregulated miRNAs play an important role in the pathogenesis of HLF [43]. For example, Li P et al. have demonstrated that miR-10396-3p is significantly decreased in the mechanical stress (MS)-induced HLF and overexpressing miR-10396-3p inhibits MS-induced HLF by targeting the inhibition expression of IL-11 [17]. Ma et al. reported that delivering the two miRNAs (miR-146a-5p and miR-221-3p) to LF cells markedly suppressed fibrosis and hypertrophy of LF in vitro and vivo [20]. Our preliminary analysis found that miR-4306 has a potential binding site with TCF7 and negatively regulates TCF7 expression in HLF cells. Previous study have shown that miR-4306 is downregulated in HLF tissues, and miR-4306 expression in HLF tissues were markedly negatively associated with the ratio of LF/spinal canal area [22]. These studies showed that miR-4306 might be involved in the pathogenesis of HLF, but the specific biological function of miR-4306 in the pathogenesis and development of HLF still remains unclear.

In the current study, we identified the TCF7 is significantly upregulated in HLF tissues and cells by integrating analysis of RNA-sequencing, bioinformatics analysis and validation experiments. Functional experiments demonstrated that TCF7 promoted hyperplasia and fibrosis of the HLF cells in vitro and in vivo. Moreover, our data demonstrated that TCF7 promoted SNAI2 expression by directly activating transcription SNAI2, and further SNAI2 inhibited miR-4306 expression by directly binding the promoter of miR-4306. Finally, our data revealed that miR-4036 negatively regulated by SNAI2 negative feedback regulated TCF7 expression by directly binding to TCF7 mRNA 3′-UTR, thus inhibiting the hyper-proliferation and fibrosis phenotype of HLF cells in vitro. Collectively, our results demonstrated not only an important role of TCF7/SNAI2/miR-4306 signaling in regulating HLF, but also provide a strong theoretical rational for developing new drugs to prevent and treat HLF.

## Materials and methods

### Human LF sample collection

This study was approved by the Institutional Research Ethics Committee of the Zhujiang Hospital of Southern Medical University. A total of 30 LF tissues samples, including hypertrophied and non-hypertrophied LF tissues, were collected from patient under-going lumbar spine surgery in Zhujiang Hospital of Southern Medical University. The hypertrophied LF tissues (> 4 mm thickness) were obtained from patient with LSCS due to LF hypertrophy, and non-hypertrophied LF tissues (≤ 4 mm thickness) were obtained from age- and gender-matched lumbar disc herniation (LDH) patient without LF hypertrophy as control. All enrolled patients were excluded from diseases such as cancer, heart disease, kidney disease, rheumatism and autoimmune diseases. All patients underwent magnetic resonance imaging scan to confirm the thickness of the LF before surgery, and all LF samples were obtained from the anatomical region (L4/5). Fibrosis score was assessed by Masson's trichrome staining according to previously report [38]. Informed consent was obtained from each patient prior to this study. Patient information in this study is summarized in Additional file 5: Table S1.

### mRNA sequencing and data analysis

Total RNAs were extracted from three independent samples of hypertrophied or non- hypertrophied LF using TRIzol reagent (Invitrogen) according to the manufacturer’s recommended protocol, and the RNA quantity was assessed with a NanoDrop ND-2000 spectrophotometer (NanoDrop Technologies). After purifying the mRNA using RiboZero Magnetic Gold Kit, the cDNA libraries were constructed for the KAPA Stranded RNA-Seq Library Prep kit (Illumina, Inc.) according to the manufacturer’s instructions. Subsequently, we used Agilent 2100 and qPCR to assess the quality and quantification of the cDNA library. Finally, the RNA-sequencing was performed by Next-Generation Sequencing with an Illumina HiSeq Xten platform. Clean data were obtained from the raw data by removing reads containing adapters, reads containing over 10% poly N, and low-quality reads, which were aligned to the specified reference genome (Homo sapiens. GRCh38, NBCI) to obtain the mapped data. The differentially expressed mRNAs between hypertrophied LF tissues and non- hypertrophied LF tissues were performed using EBseq R package. The fold changes (FCs) ≥ 2 or − 2 and false discovery rates (FDRs) < 0.05 served as the screening criteria to get differentially expressed mRNA.

### Primary LF cell culture

According to our previously described method [4], LF cells were isolated and cultured from the LF tissues of patients with LSCS or LDH. Briefly, the obtained LF samples were cut into small pieces and digested for 2 h at 37 °C using Dulbecco’s modified Eagle’s medium (DMEM, Gibco) with 0.2% type I collagenase (Gibco), then seeded on to cell culture dish and incubated with DMEM containing 10% fetal bovine serum (FBS, Gibco) and 1% penicillin/streptomycin (Invitrogen). The isolated cells were observed for fibroblast morphology and identified the expression of specific markers [collagen I (1:100, #72026, Cell Signaling Technology) and Vimentin (1:500, ab16700, Abcam)] by immunofluorescence staining. Subsequent experiments were conducted using cells from the third passage to forth passage of LF cells.

### Adenovirus construction and infection

The adenovirus of TCF7 overexpression/knockdown or SNAI2 overexpression/knockdown were constructed by Genechem (Nanjing, China). The adenovirusl particles of miR-4306 mimic/inhibitor and negative control (NC) mimics/inhibitor were obtained from Ribobio Inc. (Guangzhou, Guangdong, China). All cells were infected with the adenovirus according the manufacturer’s instructions, and the infection efficiency was verified by real-time quantitative PCR (RT-qPCR) or Western blotting analyses. All shRNA and miR-4306 mimics/inhibitor sequences are listed in Additional file 6:Table S2.

### RT-qPCR

Total RNA from the cells or tissues sample was extracted using Trizol (Invitrogen, Carlsbad, CA). Reverse transcription and RT-qPCR for miR-4306 were carried out using miRNA 1st Strand cDNA Synthesis Kit (MR101-01, Vazyme) and miRNA Universal SYBR qPCR Master Mix (MQ101-01, Vazyme) on a LightCycler^®^96 (Roche). Reverse transcription and RT-qPCR for the mRNAs were performed as described in our previous studies [4].U6 small nuclear RNA and GAPDH were used as an internal control for miR-4306 and mRNAs, respectively. The relative RNA expression of genes was calculated according to the Ct (2^−ΔΔCt^) method. All experiments were performed in triplicate. All specific primers sequence in study are listed in Additional file 7:Table S3.

### Western blotting

The nuclear protein extraction was performed using the Nuclear and Cytoplasmic Protein Extraction Kit (Beyotime, Shanghai, China) according to the protocol of manufacturer, and then subjected to western blotting analysis. Western blotting was conducted as described in our previous studies [4]. Primary antibodies antibody information is as follows: cleaved Caspase-3 (1:500, ab32042, Abcam), Bax (1:1000, ab182733, Abcam), Bcl-2 (1:500, ab182858, Abcam), TCF7 (1:1000, #2203, Cell Signaling Technology), SNAI2 (1:1000, #9585, Cell Signaling Technology), collagen I (1:1000, ab260043, Abcam), collagen III (1:1000, ab7778, Abcam), MMP2 (1:500, ab181286, Abcam), MMP13 (1:1000, ab39012, Abcam), TGF-1β (1:1000, ab215715, Abcam), and GAPDH (1:1000, ab8245, Abcam).

### Cell proliferation

Cell proliferation ability was detected using a Cell Proliferation Reagent Kit I (MTT, Sigma-Aldrich) according to the manufacturer’s recommended protocol. Absorbance values were measured at the wavelength of 490 nm. All experiments were performed in three to five biological duplicates.

### Cell apoptosis

Cells apoptosis was measured by flow cytometry using Annexin V-FITC/propidine iodide (PI) double-staining kit (Abcam) according to the manufacturer’s instructions. All experiments were performed in three biological duplicates.

### Chromatin immunoprecipitation

A chromatin immunoprecipitation (ChIP) assay was carried out using the ChIP assay kit (ab500, Abcam) according to the manufacturer’s instructions. Briefly, the following antibodies were used to immunoprecipitate cross-linked protein-DNA complexes: rabbit anti-TCF7 (1:50, #2203, Cell Signaling Technology, USA), rabbit anti-SNAI2 (1:50, #9585, Cell Signaling Technology, USA), and normal rabbit IgG (1:50, #2729, Cell Signaling Technology, USA). After cross-linked protein-DNA complexes to free DNA, the immunoprecipitated DNA was purified for RT-qPCR analyses with specific primers.

### Dual-luciferase reporter assay

For validation of transcription factor interactions with target genes, the TCF7-binding motif in the promoter region of SNAI2 and the SNAI2-binding motif in the promoter region of miR-4306 were predicted through JASPAR (http://jaspar.genereg.net/). According to the predicted results, different truncated plasmid of SNAI2 or miR-4306 promoter was co-transfected with corresponding transcription factors plasmid into 293 T cells. For the verification of interaction between miR-4306 and TCF7, the wild type or mutant type of TCF7 3′-UTR (containing the binding site of miR-4306) was cloned into the luciferase vector, and then transfected into 293 T cells together with miR-4306 mimics or the negative control (NC mimics), respectively. Transfer after 24 h, luciferase activities were assessed using a Dual Luciferase Assay Kit (Promega, Madison, WI, USA) in accordance with the manufacturer’s instructions. The relative luciferase activities were calculated by normalizing the signal value of Renilla luciferase to Firefly luciferase, and then compared with the negative control.

### RNA immunoprecipitation

RNA immunoprecipitation (RIP) experiments were performed with a Magna RIP™ RNA-Binding Protein Immunoprecipitation Kit (Millipore, Billerica, MA) according to the manufacturer’s instructions. AGO2 antibody ((Millipore, Billerica, MA, USA)) or negative control IgG antibody (Millipore, Billerica, MA, USA) was used for RIP. Co-precipitated RNAs were finally extracted with RNeasy Mini Kit (QIAGEN, China) for RT-qPCR to demonstrate the presence of the binding targets.

### Animal experiments

All animal experimental protocols were approved by the Ethics Committee for Animal Research of the Zhujiang Hospital of Southern Medical University (Guangzhou, China). All mice purchased from the Experimental Animal Center of Southern Medical University were 8-week-old C57BL/6 male mice. The mice model with HLF was constructed by the hydrophobic characteristics of mice according to the previously described [45, 46]. In brief, the mice were placed in a beaker with 5 mm of animal water at the bottom to induce a bipedal standing posture. The mice were maintained in a bipedal standing posture for 6 h a day with an interval of 2 h of free activity, and the above operations were continued for 8 weeks to construct the HLF mice model. The control mice were placed in a same condition as the mice with HLF model except for the bottom without animal water. To verify the therapeutic effect of silencing TCF7 on the mouse HLF model, 20 mice were randomly assigned to the control group, HLF model group, HLF + AAV-shNC group, and HLF + AAV-shTCF7 group. After six weeks of bipedal induction, we made a longitudinal skin incision in the mouse lumbar spine, and removed the dorsal paravertebral muscle from the spinous processes and laminae to expose the L5/6 LF under a surgical microscope, thereby AAV-shNC or AAV-shTCF7 (1 × 10^12^ vg/ml, 3 µl) from Hanbio Biotechnology (Shanghai, China) was injected into L5/6 LF in anesthetized mice with a microinjector (NF36BV 36GA, NanoFil, United States). After 4 weeks of AVV injection, mice were euthanized and L5/6 vertebrae were collected to obtain LF tissue for later histological analyses and molecular biological analyses. Histological analyses were performed to measure the area of the LF.

### Histological analyses

hematoxylin and eosin (H&E) staining and Elastica van Gieson (EVG) staining were performed to measure the area of the LF and degree of LF fibrosis, respectively. The LF samples from mice or humans were fixed overnight with 4% paraformaldehyde (Beyotime, shanghai, China), paraffin-embedded, and cut into slices (4 μm). After dewaxing and dehydration, the slices were performed by H&E kit (Beyotime, shanghai, China) or EVG kit (JianChen, Nanjing, China) according to the manufacturer’s instructions. Quantitative analyses of the LF areas and degree of LF fibrosis (the ratio of elastic fibers to collagen fibers) were obtained using ImageJ software (NIH, United States). Each sample was detected three times, and the average value was taken.

### Immunohistochemistry

Immunohistochemical staining was conducted using Histostain®-SP Kits according to the manufacturer’s instructions. In brief, the LF samples from humans were fixed overnight with 4% paraformaldehyde (Beyotime, shanghai, China), paraffin-embedded, and then cut into slices with 4 μm thickness. After dewaxing and antigen retrieval, the slices were organization background closed using serum blocking reagent. Then these slices were incubated with TCF7 primary antibodies (1:200, #2203, Cell Signaling Technology) and β-catenin (1:100, ab16051, Abcam) overnight at 4 °C. Subsequently, these sections were incubated with the respective secondary antibodies (Proteintech) at room temperature. Immunohistochemical results were visualized using a Zeiss microscope (Carl Zeiss Meditec AG, Jena, Germany) and then analyzed by ImageJ software (NIH, United States).

### Statistical analysis

Results are presented as the means ± SD. All the statistical analyses were performed using the GraphPad Prism 6.0 (GraphPad Software, La Jolla, USA). Statistical significance for comparisons between two groups were analyzed using Student’s t-test, and statistical significance for comparisons among more than two group was analyzed using one-way ANOVA. The p < 0.05 was considered as significant difference.

## Results

### High expression of TCF7 in HLF tissues and cells

**Fig. 1:**
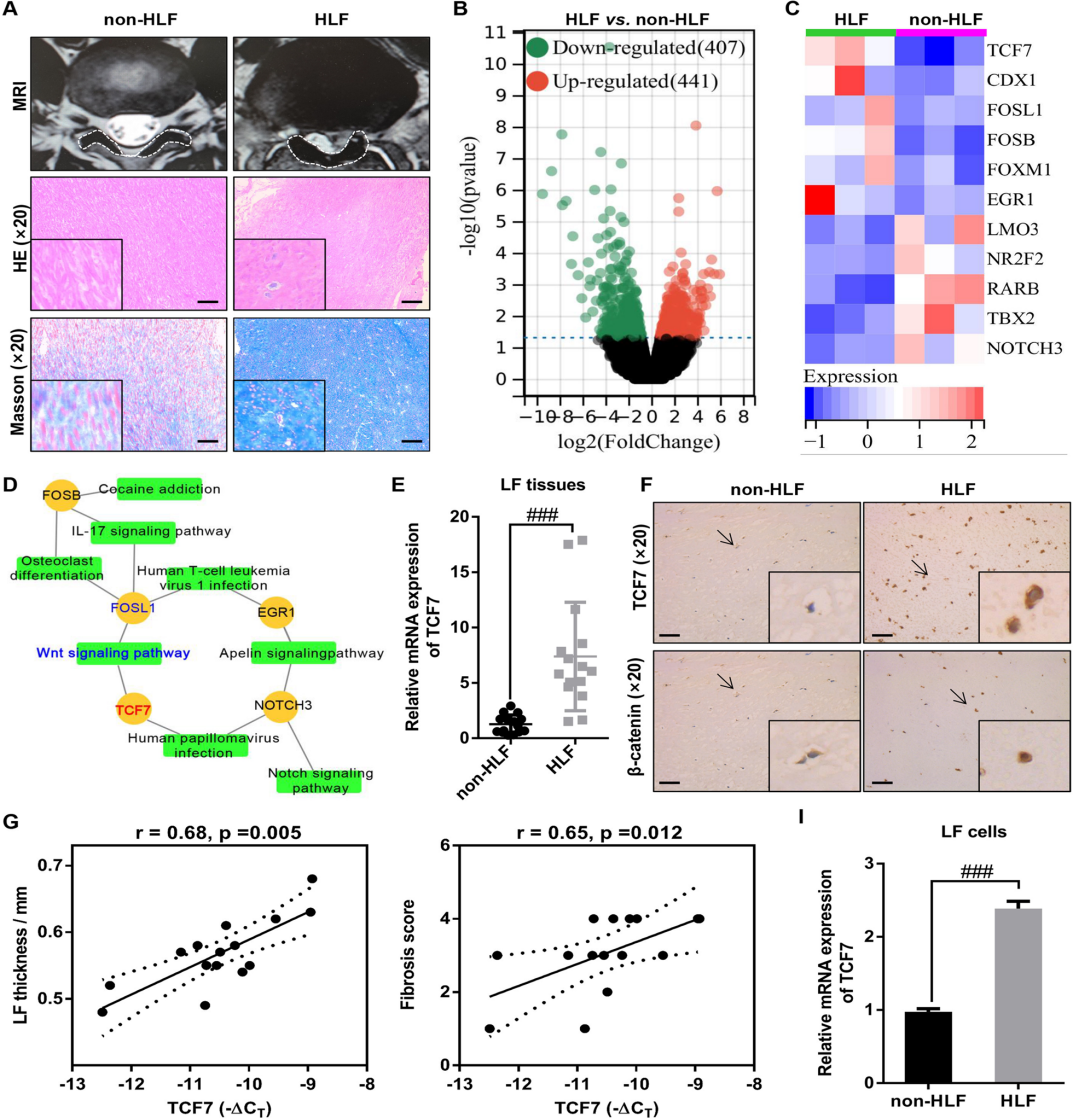
High expression of TCF7 in HLF tissues and cells. **A** The HLF was determined by MRI and histopathology. Measurement of the LF thickness by MRI; Representative images of H&E staining with elastic fibers stained crimson and collagen fibers stained pink; Representative images of Masson’s trichrome staining with elastic fibers stained red and collagen fibers stained blue. Scale bar = 100 μm. **B** Volcano plot of RNA-seq data. The vertical lines correspond to 2.0-fold increase and decrease, whereas the horizontal line represents a *p*-value of 0.05. **C** Heat maps show the expression levels of differential transcription factors in HLF tissues and non-HLF tissues. **D** KEGG pathway enriched by transcription factor was visualized by Cytoscape. **E** RT-qPCR showed that the mRNA expression of TCF7 in human HLF tissue was significantly higher than that in human non-HLF tissue (n = 15). **F** Representative images of TCF7 and β-catenin immunohistochemical staining of the non-HLF tissues and HLF tissues. Scale bar = 100 μm. **G** Spearman correlation analysis showed the TCF7 expression level exhibited markedly positive correlations with the LF thickness and fibrosis score in HLF tissues. **I** RT-qPCR showed that the TCF7 expression in HLF cells was significantly higher than that in non-HLF cells. LF, Ligamentum flavum; HLF, Hypertrophy of ligamentum flavum; non-HLF, Non-hypertrophy of ligamentum flavum. ^###^P < 0.001

To explore the underlying mechanism of HLF, the mRNA sequencing was performed by in the HLF tissues and non-HLF tissues. HLF tissue or non-HLF tissue has been identified by MRI and histopathology (Fig. 1A). The results of RNA sequencing analysis showed that a total of 848 mRNAs were differentially expressed between the HLF tissues and non-HLF tissues, of which 407 were up-regulated and 441 down-regulated in HLF tissues (Fig. 1B). Then we intersected the differential mRNA with a currently known transcription factor, and found that the six transcription factor (TCF7/CDX1/FOSL1/FOSB/FOXM1/EGR1) in HLF tissues significantly higher than those in non-HLF tissues, and five transcription factor (LMO3/NR2F2/RARB/TBX2/NOTCH3) significantly downregulated (Fig. 1C). Subsequently, KEGG analysis of the above differential transcription factors showed that they were mainly enriched in WNT signaling pathway, Notch signaling pathway, and IL-17 signaling pathway (Fig. 1D). The Wnt/β-catenin pathway signaling has been shown to propagates the initiation and progression of HLF [22], and TCF7 is essential for the typical WNT/β-catenin pathway [44]. However, the relationship between TCF7 and HLF pathogenesis is unclear and needs further study. The clinical samples were expanded to verify the mRNA expression of TCF7 in the above pathways in the non-HLF tissues and HLF tissues. The results of the RT-qPCR indicated that the mRNA expression of TCF7 in HLF tissue was significantly higher than that in non-HLF tissue (Fig. 1E), which was consistent with the mRNA sequencing results. In addition, the results of the IHC also demonstrated that the protein expression of TCF7 and β-catenin in HLF tissues was significantly increased compared with that of in non-HLF tissue (Fig. 1F). Moreover, the correlation analysis revealed that the mRNA expression of TCF7 were significantly positively correlation with LF thickness and fibrosis score (Fig. 1G). In addition, the cells isolated from the HLF tissues or non-HLF tissues were identified as LF cells by the observation of cell morphology and immunofluorescence detection of LF makers expression (Additional file 1: Fig. S1), and the results showed that the isolated cells had a typical LF cells phenotype and uniformly expressed of LF cells markers (collagen I and Vimentin), suggesting that the isolated cells is the high purity of the LF cells. Then the mRNA expression of TCF7 was measured in HLF cells and non-HLF cells by RT-qPCR, and the results indicated that TCF7 expression was significantly increased in HLF cells compared with that of in non-HLF cells (Fig. 1I). Together, these data indicated that TCF7 was markedly upregulated in HLF tissue and cells, and increased TCF7 in HLF tissues associated with LF thickness and fibrosis.

### TCF7 promotes the proliferation and fibrosis of HLF cells in vitro

**Fig. 2:**
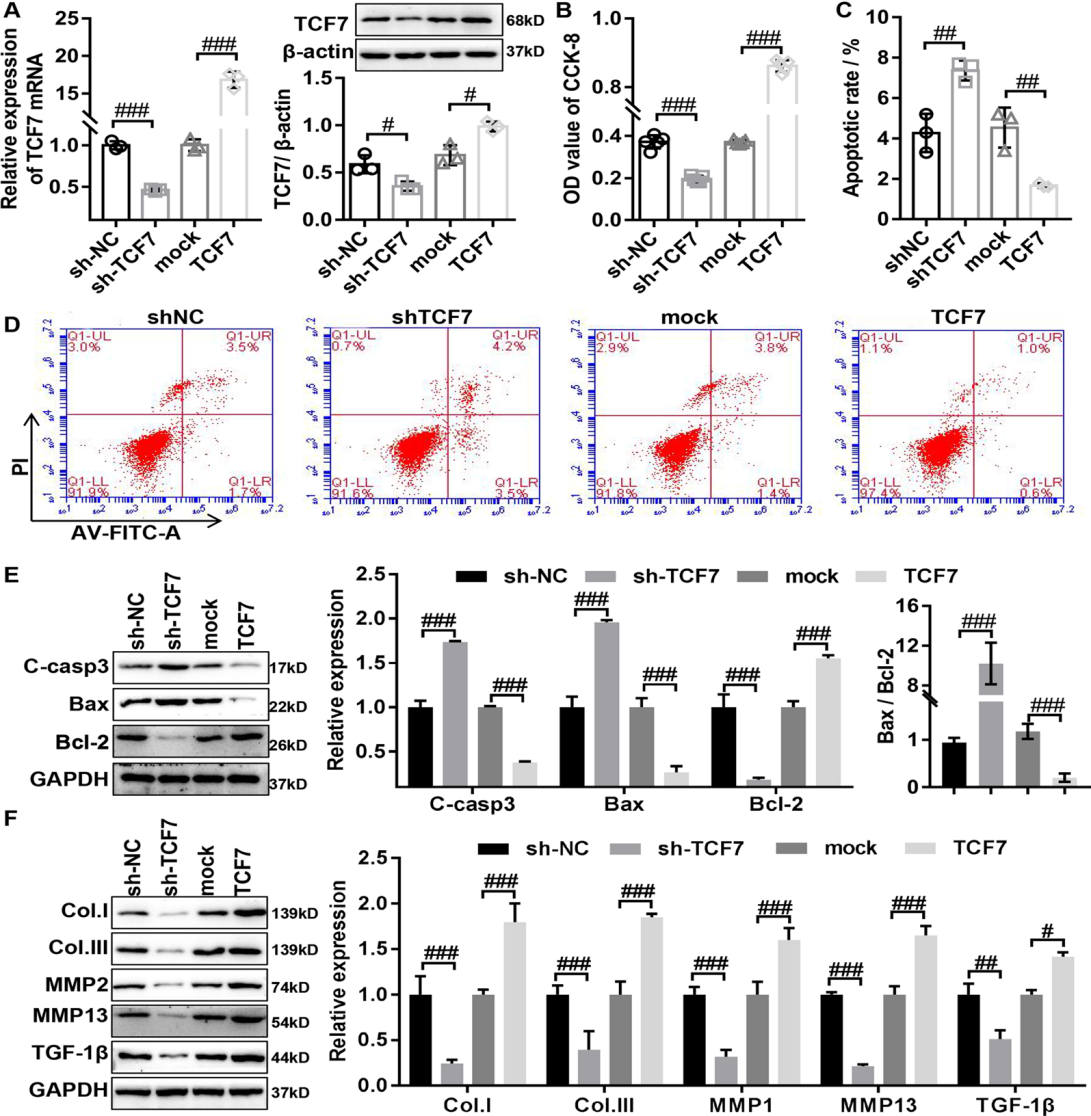
TCF7 promotes the proliferation and fibrosis of HLF cells in vitro. **A** The transfection effect of TCF7 overexpression or knockdown in HLF cells was confirmed by RT-qPCR and Western Blot. **B** The effect of overexpression or knockdown of TCF7 on the proliferation of HLF cells was detected by CCK-8 assay. **C**, **D** The effect of overexpression or knockdown of TCF7 on the apoptosis of HLF cells was assessed by flow cytometry assay. **E** The influence of overexpression or knockdown of TCF7 on the protein expression of apoptosis-related genes (cleaved caspase3, Bax, and Bcl-2) in HLF cells was confirmed by Western Blot. **F** The influence of overexpression or knockdown of TCF7 on the protein expression of fibrosis-related genes (collagen I, collagen III, MMP2, MMP13, and TGFβ1) in HLF cells was confirmed by Western Blot. C-casp3, cleaved-caspase3; col.I, collagen I; col.III, collagen III. shNC, corresponding negative control of TCF7 knockdown adenovirus; shTCF7, TCF7 knockdown adenovirus; mock, corresponding negative control of TCF7 overexpressed adenovirus TCF7; TCF7 overexpressed adenovirus. ^#^P < 0.05, ^##^P < 0.01, and ^###^P < 0.001

To explore whether TCF7 participates in the biological function of HLF cells, we analyzed the effect of overexpressing or silencing TCF7 (Fig. 2A) on viability, apoptosis, and fibrosis in HLF cells. The cell viability was detected using the CCK-8 assay, and the results showed that overexpression of TCF7 significantly promoted viability of HLF cells, whereas knockdown of TCF7 resulted in the opposite result (Fig. 2B). Then cell apoptosis was measured by using the flow cytometry and western blot. The results of flow cytometry indicated that overexpressing TCF7 inhibited apoptosis ratios of HLF cells, whereas knockdown of TCF7 promoted apoptosis ratios of HLF cells (Fig. 2C and D). Consistent with the results of flow cytometry, the results of western blot demonstrated that overexpression of TCF7 in HLF cells involved in inhibition of Bax and cleaved caspase-3 protein expression and activation of Bcl-2 protein expression, whereas knockdown of TCF7 resulted in the opposite result (Fig. 2E). Furthermore, to confirmed the relationship between the TCF7 expression and fibrosis in HLF cells, western blot was used to assess the effect of overexpressing or silencing TCF7 on the expression of fibrosis-related proteins (Collagen I, Collagen III, MMP13, MMP2, and TGF-β1) in HLF cells. The results of western blot revealed that overexpression or knockdown of TCF7 enhanced or suppressed the expression of fibrosis-related proteins (Collagen I, Collagen III, MMP13, MMP2, and TGFβ), respectively (Fig. 2F), suggesting TCF7 promoted fibrogenesis in HLF cells. Together, the above data demonstrated that TCF7 enhanced the proliferation, anti-apoptosis, and fibrosis in HLF cells.

### TCF7 knockdown inhibited hypertrophy and fibrosis of LF in vivo

**Fig. 3:**
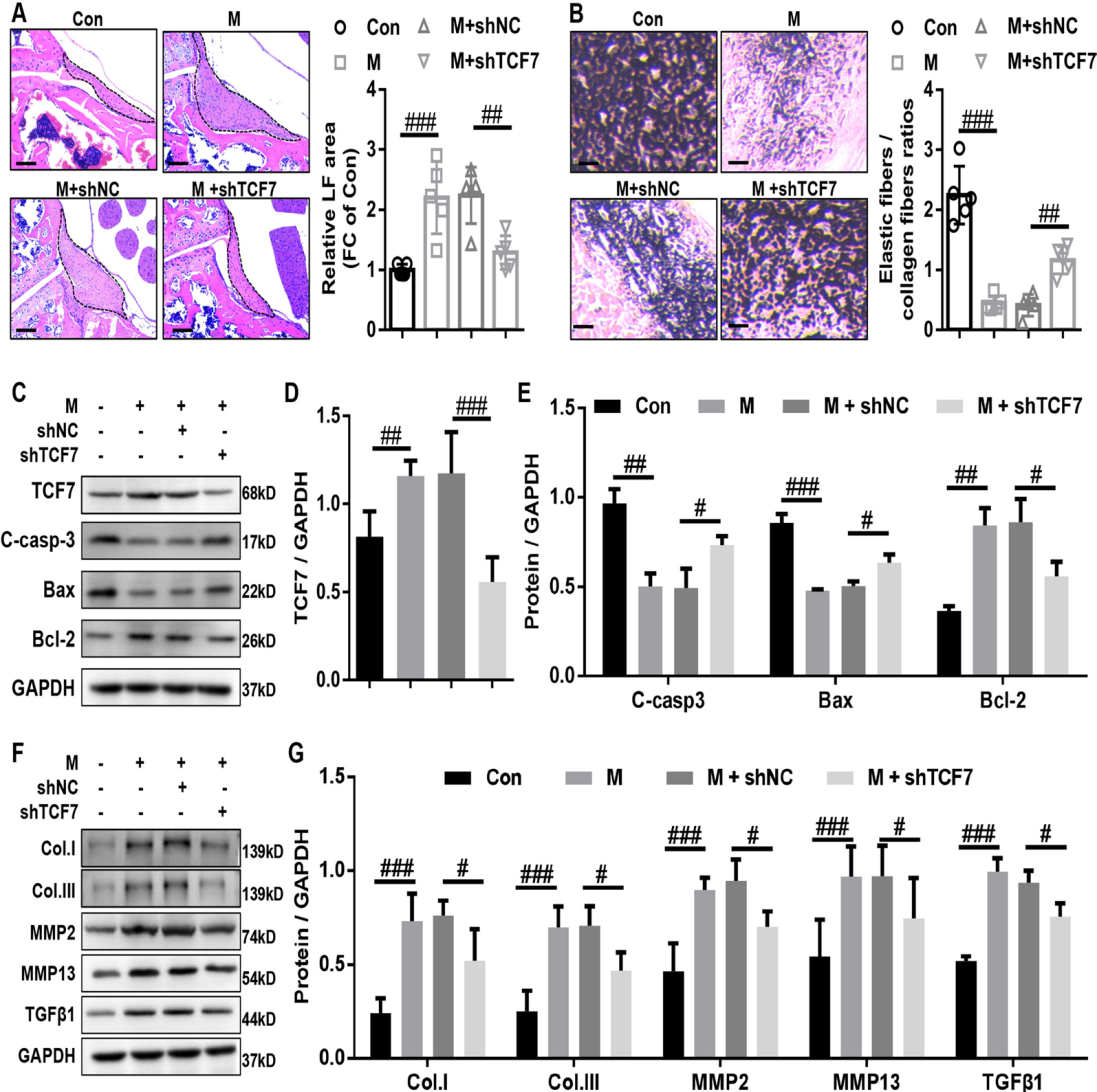
TCF7 knockdown inhibited hypertrophy and fibrosis of LF in vivo. **A** Representative images of H&E staining of the LF and quantitative analysis of the LF area. Scale bar = 100 μm. **B** Representative images of EVG staining of the LF and quantitative analysis of the ratio of elastic fibers area to collagen fibers area. Scale bar = 20 μm. **C**, **D** Effect of TCF7 knockdown on the protein expression of TCF7 in mice with LF tissues as measured by Western Blot. **C**, **E** Effect of TCF7 knockdown on the protein expression of apoptosis-related genes (cleaved caspase3, Bax, and Bcl-2) in mice with LF tissues as measured by Western Blot. **F**, **G** Effect of TCF7 knockdown on the protein expression of fibrosis-related genes (collagen I, collagen III, MMP2, MMP13, and TGFβ1) in mice with LF tissues as measured by Western Blot. Con, control; M, mice with ligamentum flavum hypertrophy induced by bipedal standing; shNC, corresponding negative control of TCF7 knockdown adenovirus; shTCF7, TCF7 knockdown adenovirus. ^#^P < 0.05, ^##^P < 0.01, and ^###^P < 0.001

Current studies have shown that the AAV vector system is a promising delivery vehicle of gene therapy because of its safe and long-term efficacy [23, 33]. In order to further confirm the role of the TCF7 in LF, we evaluated the therapeutic effect of TCF7 knockdown on mice with HLF by in situ injection of AAV-shNC or AAV-shTCF7. The results of HE staining and EVG staining indicated that LF area and the ratio of collagen fibers to elastic fibers significantly increased in the bipedal standing-induced HLF mice (Fig. 3A and B), which were consistent with the previous studies in humans and animals [30, 45, 46], suggesting that the mouse HLF model was successfully constructed in the study. The results of western blot demonstrated that the administration of the AAV-shNC had no effect on the high expression of TCF7 in mouse HLF tissues, while the administration of the AAV-shTCF7 significantly inhibited TCF7 expression in mouse HLF tissues (Fig. 3C and D). Further our data showed that no significant difference was observed in the LF area between the HLF mouse and HLF mouse treated with AAV-shNC (Fig. 3A and B), indicating that the administration of the AAV-shNC had no effect on HLF. Compared with the HLF mouse treated with AAV-shNC, the administration of the AAV-shTCF7 obviously decreased LF area and the ratio of collagen fibers to elastic fibers (Fig. 3A and B), suggesting that administration of the AAV-shTCF7 inhibited hypertrophy and fibrosis of LF induced by bipedal standing posture in mice. Moreover, the expression levels of apoptosis-related proteins (Bax, Bcl-2, and cleaved caspase-3) and fibrosis-related proteins (Collagen I, Collagen III, MMP13, MMP2, and TGFβ1) were detected by western blot in LF tissues of mice. The results indicated that the protein expression of the Bax and cleaved caspase-3 was decreased and the protein expression of Bcl-2, Collagen I, Collagen III, MMP13, MMP2, and TGFβ1 was increased in mice with HLF compared with that of in mice with non-HLF, and the above phenomena were reversed by the administration of the AAV2-shTCF7 (Fig. 3C, E, F, and G). Together, our data revealed that TCF7 knockdown could restrain the hypertrophy and fibrosis of LF in bipedal standing mouse model.

### TCF7 promote the cell proliferation and fibrosis through activation of SNAI2 transcription in HLF cells

**Fig. 4:**
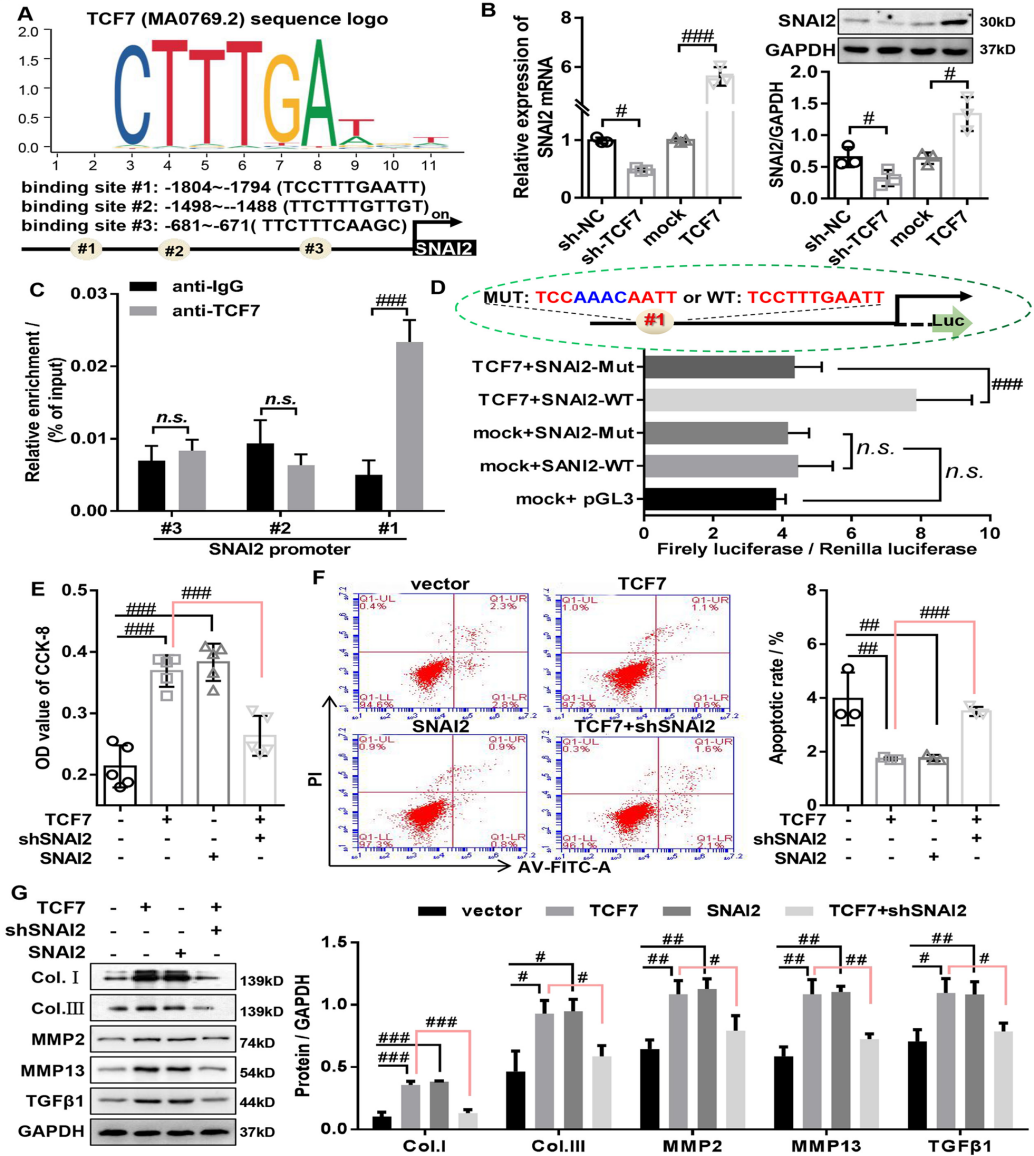
TCF7 promotes the cell proliferation and fibrosis through activation of SNAI2 transcription in HLF cells. **A** JASPAR predicted the DNA binding motif of TCF7 and the possible binding sites of TCF7 in SNAI2 promoter. **B** The mRNA and protein expression of SNAI2 was evaluated by RT-qPCR and Western Blot in HLF cells with TCF7 knockdown or overexpression. **C** ChIP assays validated the interaction between SNAI2 promoter and TCF7. **D** Luciferase reporter assays confirmed that TCF7 bound to SNAI2 promoter at the putative sites. **E** The proliferation of HLF cells transfected with vector, TCF7, SNAI2, and TCF7 + shSNAI2 was determined by CCK-8 assays. **F** The apoptosis of HLF cells transfected with vector, TCF7, SNAI2, and TCF7 + shSNAI2 was determined by flow cytometry assays. **G** Western Blot was used to detect the protein expression of fibrosis-related genes (collagen I, collagen III, MMP2, MMP13, and TGFβ1) in HLF cells transfected with vector, TCF7, SNAI2, and TCF7 + shSNAI2. shSNAI2, SNAI2 knockdown adenovirus; SNAI2, SNAI2 overexpressed adenovirus; TCF7 overexpressed adenovirus. ^#^P < 0.05, ^##^P < 0.01, and ^###^P < 0.001

To further elucidate the underlying mechanism by which TCF7 promotes LF fibrosis, the TRRUST ver.7.3 (https://www.grnpedia.org/trrust/result.php) database and JASPAR database (https://jaspar.genereg.net/search?advanced=true) were used to predict the promoters of the SNAI2 genes. The results showed that promoter region of the SNAI2 contained three TCF7 binding sites (Fig. 4A). While the results of RT-qPCR and western blot showed that overexpressing TCF7 markedly increased the mRNA and protein expression of SNAI2 in HLF cells, while silencing TCF7 displayed the opposite effects (Fig. 4B). These results implied that TCF7 might bind to the SNAI2 promoter to positively regulate SNAI2 expression in HLF cells. ChIP assay confirmed that TCF7 can directly associate with the SNAI2 promoters, and observed successful recruitment of TCF7 by binding sites #1 rather than binding sites #2 and #3 (Fig. 4C). Consistently, the results of luciferase reporter assay showed that overexpressing TCF7 significantly increased SNAI2 promoter-driven reporter activity HLF cells (Fig. 4D), further confirming TCF7 directly targets SNAI2 and activates its transcription expression.

Next, we explored whether the overexpression of TCF7 enhanced the proliferation and fibrosis by activation of SNAI2 in HLF cells. The increased viability of HLF cells induced by overexpressing TCF7 was also dramatically abrogated by the silencing SNAI2 (Additional file 2: Fig. S2A and Fig. 4E). Furthermore, the results of flow cytometry showed that silencing SNAI2 significantly decreased the inhibitory effect of TCF7 overexpression on the apoptosis rate (Fig. 4F). Consistently, the results of western blot indicated that silencing SNAI2 significantly reversed the effect of TCF7 overexpression on the protein expression of apoptosis-related markers (Bax, cleaved caspase-3, and Bcl-2) in HLF cells (Additional file 2: Fig. S2B). These results demonstrated that TCF7 inhibited apoptosis of HLF cells by upregulating SNAI2. Furthermore, the increased protein expression of fibrosis-related proteins (Collagen I, Collagen III, MMP13, MMP2, and TGFβ1) induced by overexpressing TCF7 was also markedly reversed by the silencing SNAI2 (Fig. 4G). Moreover, our data also showed that overexpressing SNAI2 significantly reversed the inhibition effect of TCF7 knockdown on the cell viability, anti-apoptosis, and the protein expression of fibrosis-related markers (Additional file 3: Fig. S3), indicating that silencing TCF7 inhibit the proliferation and fibrosis of HLF cells by downregulating SNAI2. Interestingly, we also found that the overexpressing SNAI2 in HLF cells significantly enhanced the cell viability, anti-apoptosis, and the protein expression of fibrosis-related markers (Additional file 3: Fig. S3 and Fig. 4E–G). Whereas silencing SNAI2 in HLF cells remarkably decreased the cell viability, anti-apoptosis, and the protein expression of fibrosis-related markers compared with vector group (Additional file 3: Fig. S3). Together, our data suggested that activation of SNAI2 contributes to TCF7-mediated LF hypertrophic and fibrotic phenotype in vitro.

### SNAI2/miR-4306 axis negative feedback regulates TCF7 expression

**Fig. 5:**
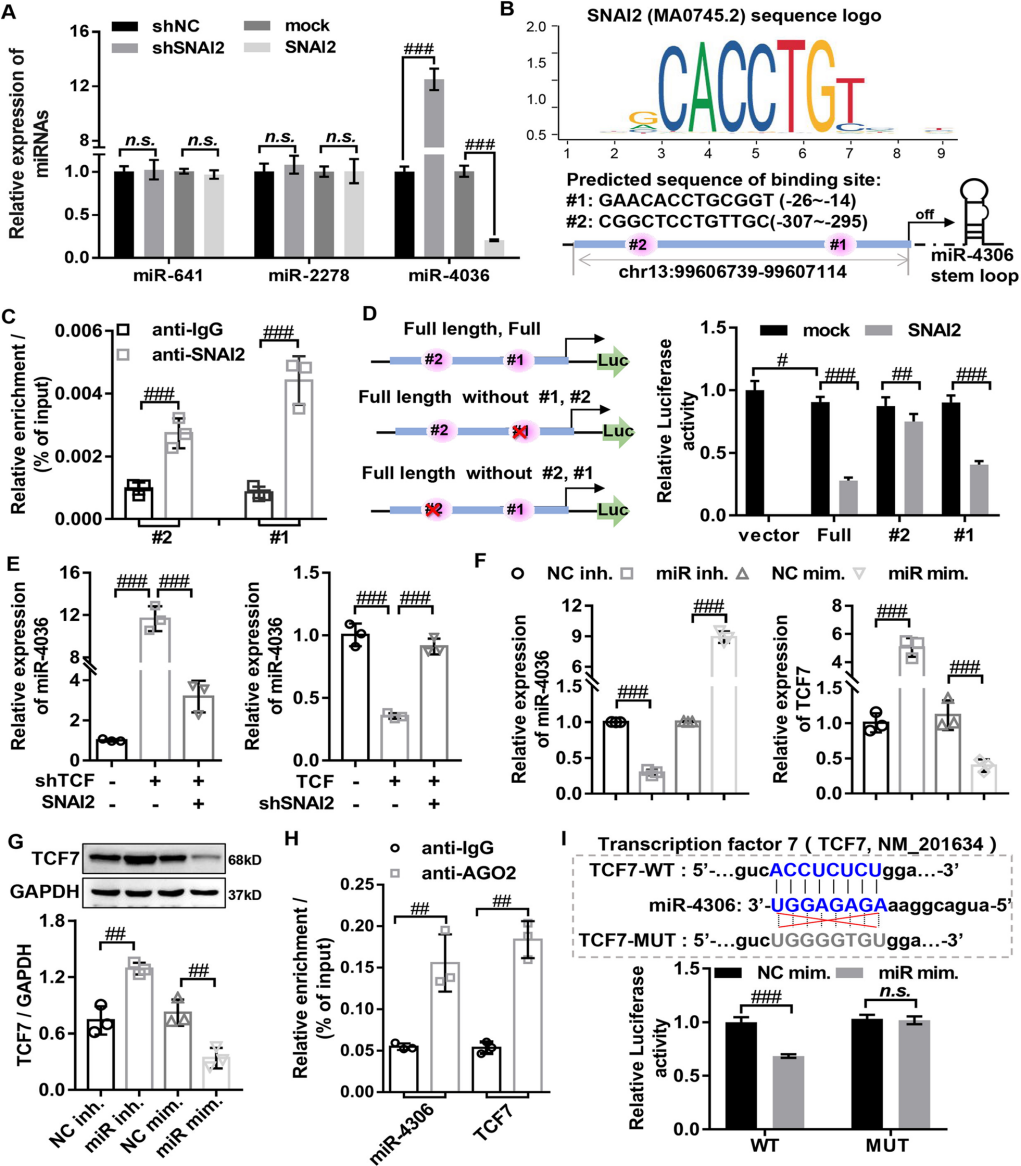
SNAI2/miR-4306 axis negative feedback regulates TCF7 expression. **A** The expression of miRNAs (miR-4306, miR-641 and miR-2278) was evaluated by RT-qPCR in HLF cells with SNAI2 knockdown or overexpression. **B** JASPAR predicted the DNA binding motif of SNAI2 and the possible binding sites of SNAI2 in miR-4306 promoter. **C** ChIP assays validated the interaction between miR-4306 promoter and SNAI2. **D** Luciferase reporter assays confirmed that SNAI2 bound to miR-4306 promoter at the putative sites. **E** RT-qPCR used to detect the miR-4306 expression in HLF cells transfected with vector, shTCF7, shTCF7 + SNAI2, TCF7, and TCF7 + shSNAI2. **F** The expression of miR-4306 and TCF7 was evaluated by RT-qPCR in HLF cells with miR-4306 knockdown or overexpression. **G** The protein expression of TCF7 was evaluated by Western Blot in HLF cells with miR-4306 knockdown or overexpression. **H** RIP assays demonstrated the interaction between miR-4306 and TCF7. **I** Predicted miR-4306 target sequence in TCF7-3′ UTR (TCF7-WT) and mutant containing mutated nucleotides in the seed sequence of TCF7-3′ UTR (TCF7-Mut), and the activity of the TCF7-3′ UTR-WT and TCF7-3′ UTR-Mut luciferase reporter gene in indicated cells. shSNAI2, SNAI2 knockdown adenovirus; shNC, corresponding negative control of SNAI2 knockdown adenovirus; SNAI2, SNAI2 overexpressed adenovirus; mock, corresponding negative control of SNAI2 overexpressed adenovirus; shTCF7, TCF7 knockdown adenovirus; TCF7 overexpressed adenovirus; NC inhi., negative control inhibitor; miR inhi., miR-4306 inhibitor; NC mim., negative control mimics; miR mim., miR-4306 mimics; ^#^P < 0.05, ^##^P < 0.01, and ^###^P < 0.001

As shown in Fig. 4 and Additional file 3: Fig. S3, SNAI2 is involved in the malignant biological phenotype of HLF cells, such as abnormal proliferation and fibrosis. However, the molecular mechanism by which SNAI2 regulated the malignant biological phenotype of HLF cells remained unclear. As a transcription factor, SNAI2 could inhibit the transcription by directly binding to miRNAs promoters [8, 9, 34]. The TransmiR v2.0 database (http://www.cuilab.cn/transmir) database was used to predict miRNAs that motif of SNAI2 binds to miRNAs promoters, and found that motif of SNAI2 had binding sites with the promoters of three miRNAs (miR-4306, miR-641, and miR-2278). The results of RT-qPCR showed that overexpressing or silencing SNAI2 markedly decreased or increased the miR-4306 expression in HLF cells, but the expression levels of miR-641 and miR-2278 were not regulated by SNAI2 (Fig. 5A). These data suggested that SNAI2 might bind to miR-4306 promoter to negatively regulate miR-4306 expression in HLF cells. To confirmed the binding regions of miR-4306 promoter regulated by SNAI2, two binding sites for SNAI2 motif on miR-4306 promoter region were predicted by the JASPAR database (Fig. 5B). ChIP assay demonstrated that SNAI2 could bind the promoter of miR-4306 directly, and observed successful recruitment of SNAI2 by binding sites #1 or site #2, and the effect of binding site #1 was better than binding site #2 (Fig. 5C). Consistently, the results of luciferase reporter assay showed that overexpressing SNIAI2 significantly decreased miR-4306 promoter-driven reporter activity (Fig. 5D), further confirming SNIAI2 directly targets miR-4306 and activates its transcription expression. Importantly, we found that overexpressing or silencing TCF7 markedly decreased or increased miR-4306 expression, the phenomenon was dramatically abrogated by the silencing or overexpressing SNAI2 (Fig. 5E), suggesting that TCF7 negatively regulated miR-4306 expression by mediating SNAI2 expression. Together, these data demonstrated that SNAI2 positively regulated by TCF7 inhibited the transcription of miR-4306 by binding to the promoter region of miR-4306.

Previous studies have shown that TCF7 is negatively regulated by miRNAs [12, 28]. To explore whether miR-4306 negatively regulated TCF7 expression in HLF cells, the RAID and miRDB database were used to predict the interaction between TCF7 and miR-4306. The results showed that TCF7 3’UTR had potential binding sites with miR-4306. And the overexpressing miR-4306 markedly inhibited the mRNA and protein expression of TCF7 in HLF cells, while silencing miR-4306 displayed the opposite effect (Fig. 5F and G). these results suggested that TCF7 may be a target gene of miR-4306 in HLF cells. The relationship between miR-4306 and TCF7 was evaluated through RIP experiments, the results revealed the interaction between miR-4306 and TCF7 (Fig. 5H). Further dual-luciferase reporter assay was used to confirm the specific binding site of miR-4306 and TCF7. The sequence of TCF7 3′-UTR including the miR-4306 binding site (TCF7 3′-UTR-WT) or its corresponding mutation sequence (TCF7 3′-UTR-MTU) was inserted into the psiTM-Check2 luciferase vector, and then co-transfected 293 T cells with NC mimics or miR-4306 mimic and TCF7 3′-UTR-WT plasmids or TCF7 3′-UTR-MTU plasmids, respectively. The results showed that miR-4306 overexpression greatly inhibited the luciferase activity of TCF7 3′-UTR-WT reporter, but luciferase activity of TCF7 3′-UTR-MUT was not significantly impacted by miR-4306 overexpression (Fig. 5I), indicating that miR4306 directly binds to the TCF7 3′-UTR region to regulate TCF7 transcription. In summary, our data demonstrated that SNAI2 enhanced TCF7 expression by transcriptionally inhibiting miR-4306 expression.

### The miR-4306 negatively regulated by SNAI2 inhibited the proliferation and fibrosis of HLF cells in vitro

**Fig. 6:**
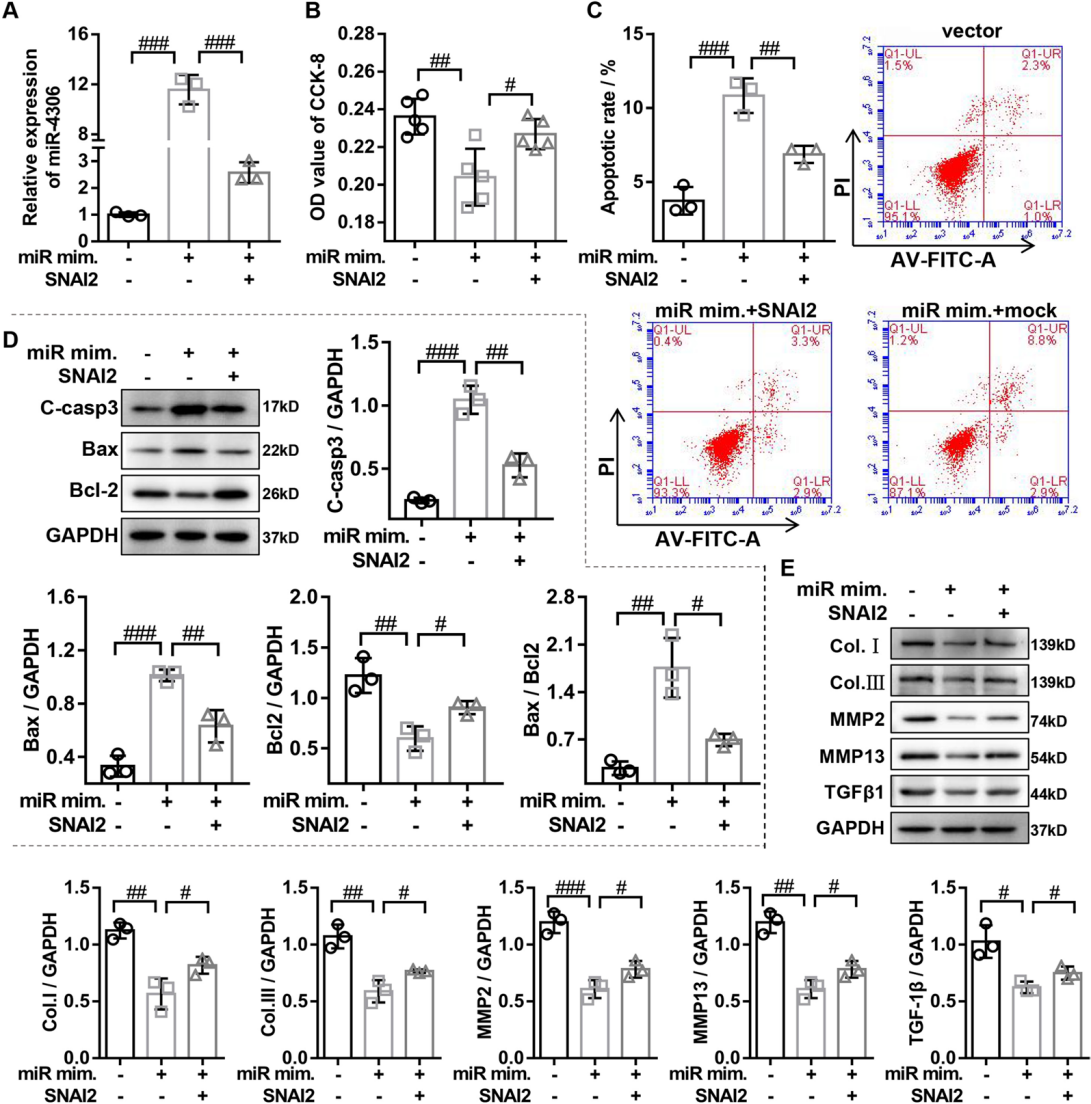
The miR-4306 negatively regulated by SNAI2 inhibited the proliferation and fibrosis of HLF cells in vitro. **A** RT-qPCR was used to detect the miR-4306 expression in HLF cells transfected with vector, miR-4306 mimics, and miR-4306 mimics + SNAI2. **B** The proliferation of HLF cells transfected with vector, miR-4306 mimics, and miR-4306 mimics + SNAI2 was determined by CCK-8 assays. **C** The apoptosis of HLF cells transfected with vector, miR-4306 mimics, and miR-4306 mimics + SNAI2 was determined by flow cytometry assays. **D** Western Blot was used to detect the protein expression of apoptosis-related genes (cleaved caspase3, Bax, and Bcl-2) in HLF cells transfected with vector, miR-4306 mimics, and miR-4306 mimics + SNAI2. **E** Western Blot was used to detect the protein expression of fibrosis-related genes (collagen I, collagen III, MMP2, MMP13, and TCFβ1) in HLF cells transfected with vector, miR-4306 mimics, and miR-4306 mimics + SNAI2. miR mim., miR-4306 mimics; SNAI2, SNAI2 overexpressed adenovirus. ^#^P < 0.05, ^##^P < 0.01, and ^###^P < 0.001

**Fig. 7:**
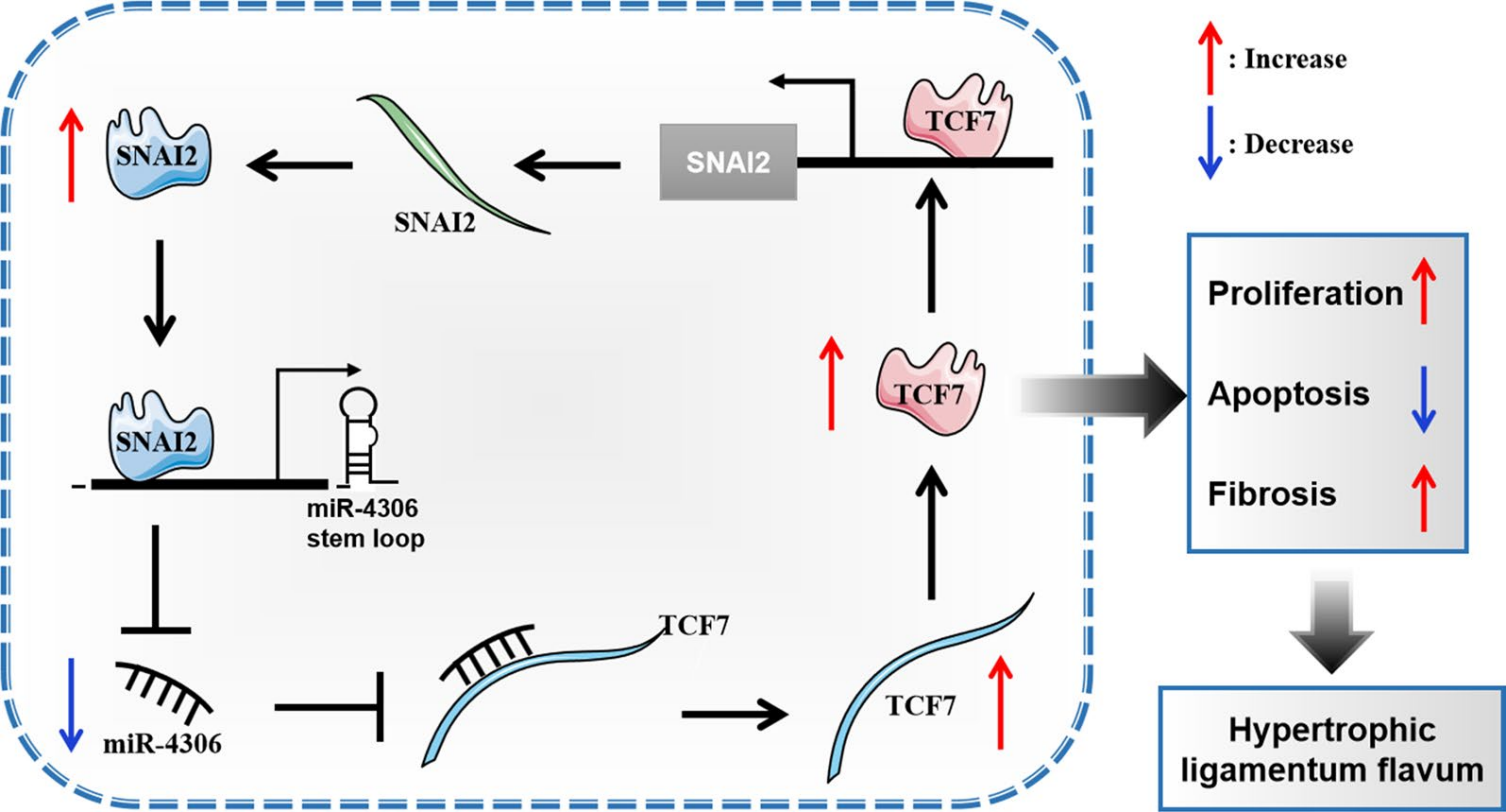
Diagram depicting the regulation mechanism of TCF7/SNAI2/miR-4306 feedback loop in ligamentum flavum hypertrophy

It has been reported that miR-4306 is significantly down-regulated in HLF tissues (fold change = − 1.26, P = 0.005), and its expression level was significantly negatively correlated with LF/spinal canal area ratio (LSAR), suggesting that miR-4306 may be involved in the occurrence and development of HLF [22]. However, the role of miR-4306 in HLF cells has not been explored. HLF cells were transfected with miR-4306 mimics or miR-4306 inhibitor to evaluate the effects of miR-4306 on the viability, apoptosis, and fibrosis of HLF cells. We found that overexpressing miR-4306 inhibited viability of HLF cells, this inhibition effect was reversed by overexpressing SNAI2 (Fig. 6A and B). The results of flow cytometry revealed that overexpressing miR-4306 significantly increased the apoptosis ratios of HLF cells, this effect was weakened by overexpressing SNAI2 (Fig. 7C). Consistently, the results of western blot also indicated that overexpressing miR-4306 significantly increased the protein expression of cleaved caspase-3 and Bax and markedly decreased the Bcl-2 protein expression, this phenomenon was reversed by overexpressing SNAI2 (Fig. 6D). Furthermore, the overexpressing miR-4306 significantly decreased the protein expression of fibrosis-related proteins (Collagen I, Collagen III, MMP13, MMP2, and TGFβ1), the inhibition function was also markedly reversed by overexpressing SNAI2 (Fig. 6E). In addition, our data showed that silencing miR-4306 significantly promoted cell viability, anti-apoptosis, and the protein expression of fibrosis-related markers in HLF cells, this phenomenon was weakened by silencing SNAI2 (Additional file 4: Fig. S4). In summary, miR-4036 negatively regulated by SNAI2 inhibited the proliferation and fibrosis of HLF cells.

## Discussion

Although fibrosis is considered as the key pathological feature of HLF, but internal molecular mechanisms has not been fully elucidated. Herein, we confirmed for the first time that TCF7 significantly upregulated in HLF, and the TCF7 expression was remarkably positively correlated with the thickness and fibrosis degree of LF. Moreover, we demonstrated that silencing TCF7 inhibited LF hypertrophy and fibrosis by inhibiting LF cell proliferation, promoting apoptosis, and aggravating ECM degradation in vitro and vivo. Mechanically, our data revealed that augmented TCF7 led to transcriptional activation of SNAI2, and SNAI2 inhibited the transcription of miR-4306 by binding to the promoter region of miR-4306, which in turn promoted the TCF7 expression. Most importantly, we found that inhibition of TCF7/SNAI2/miR-4306 signaling suppressed LF hypertrophy and fibrosis, indicating that targeting TCF7/SNAI2/miR-4306 signaling may be a novel strategy for the prevention and treatment of HLF.

Accumulating evidence indicates that TCF7 is a critical function downstream of the typical WNT/β-catenin signaling pathway, which is involved in biological processes of many diseases [2, 18, 44]. A recent study reported that TCF7 expression was elevated in mouse heart tissue after TAC and in cardiomyocytes treated with Ang-II, and inhibition of TCF7 suppressed the occurrence of cardiac hypertrophy [18]. Base on integrating analysis of RNA-sequencing, bioinformatics analysis and validation experiments, we identified for first time that the mRNA and protein expression of TCF7 was significantly increased in HLF tissues and cells. Correlation analysis showed that TCF7 expression had significant positive correlation with LF thickness and fibrosis score, suggesting that TCF7 involved in the HLF development. Then our data demonstrated that TCF7 overexpression significantly induced proliferation and fibrosis of HLF cells. On the contrary, the silencing TCF7 significantly suppressed proliferation and fibrosis of HLF cells in vitro and suppressed the LF fibrosis and hypertrophy in vivo. In addition to proliferation and fibrosis, apoptosis is also involved in the pathological process of HLF, but the role of apoptosis in HLF remains controversial. Several investigators have shown that activation of apoptotic pathways induce cell apoptosis in HLF tissue [6, 24]. However, some researchers have shown that apoptosis is inhibited in HLF tissue. For example, Zhou et al. revealed that inhibition of apoptosis in HLF tissue compared to non-HLF tissue, which manifested as inhibition of apoptosis marker (Bax and cleaved caspase3) expression and reduction of TUNEL-positive cell percentage in human HLF tissue, and reduction cleaved caspase3 expression in the rat model with HLF [47]. At the same time, they showed that LPAR1 significantly upregulated in HLF tissue compared with that in non-HLF tissue, overexpression of LPAR1 improved inhibited apoptosis in LF cells, whereas knockdown of LPAR1 has the opposite effect [47]. Sun et al. demonstrated that upregulated WISP1 in HLF decreased apoptosis of LF cells, which manifested as inhibition of Bax and activation of Bcl-2 [30]. Furthermore, DNMT1-mediated ACSM5 significantly downregulated in LFH tissue, and ACSM5 knockdown inhibited apoptosis of HLF cells in vitro [4]. Like these studies, our data showed that upregulated TCF7 could promoted apoptosis of HLF cells, whereas silencing TCF7 significantly promoted apoptosis of HLF cells in vitro. Thus, these results implied that TCF7 inhibition might be considered as a novel potential therapeutic strategy for HLF.

Emerging evidence has demonstrated that dysregulated miRNAs is involved in the occurrence and progression of HLF [22, 43]. Yu et al. identified that miR-221 was down-regulated in HLF tissues, and overexpressing miR-221 inhibited the expression of collagens I and collagens III by sponging TIMP-2, thus inhibiting HLF formation [36]. Sun et al. revealed that miR-21 may play an important role in HLF, upregulation of miR-21 might contribute to the HLF by promoting inflammation and fibrosis via the induction of IL-6 expression [36]. The above studies suggested that miRNAs played pivotal roles in the occurrence and development of HLF, which has aroused great attention of scholars. Notably, previous study have identified that the down-regulation of miR-4306 in HLF tissues is significantly negatively correlated with increased LF/spinal canal area ratio (LSAR) [22], but the role and underlying mechanism of miR-4306 in HLF cells has not been investigated. Herein, we found that overexpression of miR-4306 in HLF cells suppressed cell hyper-proliferation and pro-fibrosis, whereas inhibition of miR-4306 displayed the opposite effect. These results showed that miR-4306 might play an anti-hypertrophy role in HLF. Furthermore, a growing body of research has revealed that miRNA-mediated target gene expression exerted an appreciable promoting or inhibiting HLF progression [5, 17, 31, 36, 43]. We conducted bioinformatics analysis using the RAID and miRDB software and found that TCF7 contained potential binding sequences for miR-4306. The RIP assay and dual luciferase activity assay confirmed that TCF7 was a direct target for miR-4306.

Remarkably, a host of studies have shown that miRNAs expressions is regulated by transcription factors (TFs) in gene regulatory networks, and the interaction between TFs and miRNAs can precisely regulate gene expressions to maintain cell homeostasis [25]. The SNAI2 encoded by the SNAI2 gene is an evolutionarily conserved C2H2 zinc finger protein that orchestrates biological processes critical to tissue development and tumorigenesis, and its main role is to facilitate the epigenetic regulation of transcriptional programs [48]. And previous studies have demonstrated that SNAI2 interacts with miRNAs promoters to inhibit its expression and then regulates cell biological functions [8, 9, 34]. Consistent with previous studies, we identified that SNAI2 inhibited the transcription of miR-4306 by directly binding to the promoter region of miR-4306. Moreover, extensive studies have revealed that SNAI2 plays an important role in various cell proliferation and fibrosis processes [10, 11, 13, 16]. However, no studies have investigated the potential biological function of SNAI2 in HLF cells. Our results revealed that SNAI2 overexpression promoted proliferation and fibrosis of HLF cells, and the inhibition of SNAI2 suppressed proliferation and fibrosis of HLF cells, indicating SNAI2 may play pro-hypertrophy roles in LF. Further rescue experiments disclosed that silencing SNAI2 could eliminate the inhibitory effect of overexpression of miR-4306 on the proliferation and fibrosis of HLF cells. Thus, our results revealed that miR-4306 was directly inhibited at the transcriptional level by SNAI2, and thereby promoted the proliferation and fibrosis of HLF cells. In addition, we found that TCF7 directly targets SNAI2 promoter and activates its transcription expression, and activation of SNAI2 contributes to TCF7-mediated LF hypertrophic and fibrotic phenotype in vitro. Therefore, our data revealed that TCF7 promoted HLF formation through mediating the TCF7/SNAI2/miR-4306 feedback loop (Fig. 7).

## Conclusion

Taken together, our study is the first to uncovered that TCF7/SNAI2/miR-4306 feedback loop promoted HLF formation by regulating proliferation and fibrosis of HLF cells, which will expand our understanding of the pathogenesis of HLF. More importantly, both in vitro and in vivo experiments implied that interfering with TCF7 could be an effective target for the prevention and treatment of HLF formation.

## Supplementary Information

**Additional file 1: Figure S1.** Identify the ligamentum flavum (LF) cells. **A** Pictures showing the morphology of the isolated cells with 1st and 3rd passage from LF tissues. **B** Identification of the phenotype of cultured LF cells using immunofluorescence staining. Immunofluorescence staining for collagen I and Vimentin in cultured cells.

**Additional file 2: Figure S2.** TCF7 inhibits the apoptosis of HLF cells by upregulating SNAI2. **A** The mRNA and protein expression of SNAI2 was evaluated by RT-qPCR and Western Blot in HLF cells transfected with vector, TCF7, SNAI2, and TCF7 + shSNAI2. **B** Western Blot was used to detect the protein expression of apoptosis-related genes (cleaved caspase3, Bax, and Bcl-2) in HLF cells transfected with vector, TCF7, SNAI2, and TCF7 + shSNAI2. shSNAI2, SNAI2 knockdown adenovirus; SNAI2, SNAI2 overexpressed adenovirus; TCF7 overexpressed adenovirus. ^#^P < 0.05, ^##^P < 0.01, and ^###^P < 0.001.

**Additional file 3: Figure S3.** Silencing TCF7 inhibits the cell proliferation and fibrosis through downregulating SNAI2 expression in HLF cells. **A** Western Blot was used to detect the protein expression of SNAI2 in HLF cells transfected with vector, shTCF7, shSNAI2, and shTCF7 + SNAI2. **B** The proliferation of HLF cells transfected with vector, shTCF7, shSNAI2, and shTCF7 + SNAI2 was determined by CCK-8 assays. **C** The apoptosis of HLF cells transfected with vector, shTCF7, shSNAI2, and shTCF7 + SNAI2 was determined by flow cytometry assays. **D** Western Blot was used to detect the protein expression of apoptosis-related genes (cleaved-caspase3, Bax, and Bcl-2) in HLF cells transfected with vector, TCF7, SNAI2, and TCF7 + shSNAI2. **E** Western Blot was used to detect the protein expression of fibrosis-related genes (collagen I, collagen III, MMP2, MMP13, and TGFβ1) in HLF cells transfected with vector, TCF7, SNAI2, and TCF7 + shSNAI2. shSNAI2, SNAI2 knockdown adenovirus; SNAI2, SNAI2 overexpressed adenovirus; shTCF7 knockdown adenovirus. ^#^P < 0.05, ^##^P < 0.01, and ^###^P < 0.001.

**Additional file 4: Figure S4.** The SNAI2 inhibits miR-4306 expression to promote the proliferation and fibrosis of HLF cells in vitro. **A** RT-qPCR was used to detect the miR-4306 expression in HLF cells transfected with vector, miR-4306 inhibitor, and miR-4306 inhibitor + shSNAI2. **B** The proliferation of HLF cells transfected with vector, miR-4306 inhibitor, and miR-4306 inhibitor + shSNAI2 was determined by CCK-8 assays. **C** The apoptosis of HLF cells transfected with vector, miR-4306 inhibitor, and miR-4306 inhibitor + shSNAI2 was determined by flow cytometry assays. **D** Western Blot was used to detect the protein expression of apoptosis-related genes (cleaved caspase3, Bax, and Bcl-2) in HLF cells transfected with vector, miR-4306 inhibitor, and miR-4306 inhibitor + shSNAI2. **E** Western Blot was used to detect the protein expression of fibrosis-related genes (collagen I, collagen III, MMP2, MMP13, and TGFβ1) in HLF cells transfected with vector, miR-4306 inhibitor, and miR-4306 inhibitor + shSNAI2. miR inh., miR-4306 inhibitor; SNAI2, SNAI2 knockdown adenovirus. ^#^P < 0.05, ^##^P < 0.01, and ^###^P < 0.001.

**Additional file 5: Table S1.** Patients information in this study.

**Additional file 6: Table S2.** The sequences of shRNAs or miR-4306 inhibitor/mimics used in this study.

**Additional file 7: Table S3.** The sequences of all the primer in qRT-PCR.

## Data Availability

The datasets generated and/or analyzed during the current study available from the corresponding author on reasonable request.
