# Supplementary material for: TCF7/SNAI2/miR-4306 feedback loop promotes hypertrophy of ligamentum flavum
Source: J Transl Med. 2022 Oct 12;20:468. doi: 10.1186/s12967-022-03677-0 (PMC9558422; doi:10.1186/s12967-022-03677-0)
Supplement: Supplementary file 5 — Additional file 5: Table S1. Patients information in this study. [file 12967_2022_3677_MOESM5_ESM.doc]

**Additional file 5**

**Table S1** Patients information in this study.

| **Index** | HLF(N=15) | Non-HLF(N=15) | P-values |
| --- | --- | --- | --- |
| Age | 65 ± 14 | 56 ± 12 | 0.07 |
| Gender | 9 females, 6 male | 8 females, 7 male | 0.48 |
| LF thickness / cm | 0.57 ± 0.05 | 0.28 ± 0.03 | < 0.001 |
| Fibrosis score | 3.07 ± 1.03 | 2.07 ± 0.71 | 0.001 |
| Lumbar level | L4/5 | L4/5 |  |
