# Supplementary material for: TCF7/SNAI2/miR-4306 feedback loop promotes hypertrophy of ligamentum flavum
Source: J Transl Med. 2022 Oct 12;20:468. doi: 10.1186/s12967-022-03677-0 (PMC9558422; doi:10.1186/s12967-022-03677-0)
Supplement: Supplementary file 6 — Additional file 6: Table S2. The sequences of shRNAs or miR-4306 inhibitor/mimics used in this study. [file 12967_2022_3677_MOESM6_ESM.docx]

**Table S2 The sequences of shRNAs or miR-4306 inhibitor/mimics used in this study.**

| Names | Sequences (5’-3’) |
| --- | --- |
| shTCF7 (human) | CAACTCTCTCTCTACGAACAT |
| shTCF7 (mouse) | AGAAGCCAGTCATCAAGAAAC |
| shSNAI2 (human) | CCCATTCTGATGTAAAGAAAT |
| miR-4306 mimics (human) | UGGAGAGAAAGGCAGUA |
| NC mimics (human) | UUGUACUACACAAAAGUACUG |
| miR-4306 inhibitor (human) | UACUGCCUUUCUCUCCA |
| NC inhibitor (human) | CAGUACUUUUGUGUAGUACAA |
