# Supplementary material for: TCF7/SNAI2/miR-4306 feedback loop promotes hypertrophy of ligamentum flavum
Source: J Transl Med. 2022 Oct 12;20:468. doi: 10.1186/s12967-022-03677-0 (PMC9558422; doi:10.1186/s12967-022-03677-0)
Supplement: Supplementary file 7 — Additional file 7: Table S3. The sequences of all the primer in qRT-PCR. [file 12967_2022_3677_MOESM7_ESM.docx]

**Table S3 The sequences of all the primer in qRT-PCR.**

| Names | Primer sequences (5’-3’) |
| --- | --- |
| TCF7 | Forward, GCGGGACAACTACGGGAAGAAG |
|  | Reverse, AGCACTGTCATCGGAAGGAACG |
| SNAI2 | Forward, GACTACCGCTGCTCCATTCCA |
|  | Reverse, TTCTGAGCCACTGTGGTCCTTG |
| GAPDH | Forward, CGGCAAGTTCAACGGCACAGT |
|  | Reverse, Reverse, ACGCCAGTAGACTCCACGACAT |
| RT primers | GTCGTATCCAGTGCAGGGTCCGAGGTATTCGCACTGGATACG ACTCAGCC |
| miR-4306 | Forward, TGGAGAGAAAGGCAGTA |
|  | Reverse, CAGTGCGTGTCGTGGAGT |
| U6 | Forward, CTCGCTTCGGCAGCACATATACT |
|  | Reverse, CAGTGCGTGTCGTGGAGT |
